# Microscopic Object Classification through Passive Motion Observations with Holographic Microscopy

**DOI:** 10.3390/life11080793

**Published:** 2021-08-05

**Authors:** Devan Rouzie, Christian Lindensmith, Jay Nadeau

**Affiliations:** 1 Department of Physics, Portland State University, 1719 SW 10th Ave., Portland, OR 97201, USA; rouzie@pdx.edu; 2 Jet Propulsion Laboratory, California Institute of Technology, Pasadena, CA 91125, USA; christian.a.lindensmith@jpl.nasa.gov

**Keywords:** holographic microscopy, Brownian motion, Strouhal, buoyancy, life detection, astrobiology, Europa, Enceladus

## Abstract

Digital holographic microscopy provides the ability to observe throughout a volume that is large compared to its resolution without the need to actively refocus to capture the entire volume. This enables simultaneous observations of large numbers of small objects within such a volume. We have constructed a microscope that can observe a volume of 0.4 µm × 0.4 µm × 1.0 µm with submicrometer resolution (in xy) and 2 µm resolution (in z) for observation of microorganisms and minerals in liquid environments on Earth and on potential planetary missions. Because environmental samples are likely to contain mixtures of inorganics and microorganisms of comparable sizes near the resolution limit of the instrument, discrimination between living and non-living objects may be difficult. The active motion of motile organisms can be used to readily distinguish them from non-motile objects (live or inorganic), but additional methods are required to distinguish non-motile organisms and inorganic objects that are of comparable size but different composition and structure. We demonstrate the use of passive motion to make this discrimination by evaluating diffusion and buoyancy characteristics of cells, styrene beads, alumina particles, and gas-filled vesicles of micron scale in the field of view.

## 1. Introduction

There is growing interest in the search for potential extant microbial life elsewhere within the Solar System, especially on the so-called Ocean Worlds such as Europa [1,2,3] and Enceladus [4,5,6]. The best methods for detecting purely microbial life are still a matter of debate, but some type of microscopy is essential for discrimination between chemistry and extant life, and a microscope (type unspecified) has been recommended for any Europa lander [7]. A perpetual issue in using microscopy for detection of new extant life is the misidentification of objects: either false positives in which non-living objects are identified as alive, or false negatives in which living objects are mistaken for abiotic particles. With optical imaging, this is particularly difficult in the 0.2 to 2 µm range, where the dimensions are in the order of a wavelength and barely resolved. Objects smaller than 0.2 µm are unlikely to be alive, or at least incapable of life in the absence of larger organisms to parasitize [8]. Larger objects are less of a problem, as they can be analyzed for sub-cellular structure and possibly even behavior.

Approaches to discriminating objects in this size range include fluorescence imaging [9] and active motility [10]. Fluorescence imaging is widely used for bacterial enumeration [11], and may be used to detect the presence of organic molecules of various types (e.g., lipids, proteins, nucleic acids) that would indicate probable biotic origin. Motility, or motion that is inconsistent with simply traveling with the background fluid or sinking/floating, is a strong indicator of likely extant life. It can also serve as a proxy for metabolism, since energy must be acquired and spent by an organism to move independently of the fluid around it. Motility can also be a response to stimuli, such as nutrients, energy sources, or toxins. Detailed characterization of microbial motility in oligotrophic environments has only begun to be performed [12,13].

Passive motion resulting from molecular collisions and gravitational effects is also important at the microbial scale; motility in some environments is energetically unfavorable since nutrients are more readily encountered via diffusion [14]. As a result, many important species are nonmotile, such as the ubiquitous marine organism *Prochlorococcus.* In this paper we develop an approach using passive vertical motion of particles in a background fluid to determine whether they are more likely to be cell-like or of mineral origin. This analysis is based upon density and the resulting time scales of Brownian motion versus sinking or floating.

Single-celled organisms that live in aqueous environments tend to have internal cellular fluids that are very similar in composition to their external medium, resulting in a density that is close to that of the external medium (on Earth this is typically water). Though limited data are available, the composition of bacterial cells is approximately 2/3 water and 1/3 other materials, where the other materials collectively have a specific gravity of about 1.3 [15]. This gives a specific gravity for a typical bacterial cell of about 1.1. Diatoms, which build a silica cell wall structure, or frustule, have a higher specific gravity of about 1.2 [16,17]. Cells of either type may regulate their buoyancy by using ion pumps on the cell membrane to change their volume or contents. Some diatoms have external structures that slow their sinking rate. Cyanobacteria are unique in being able to regulate their buoyancy by means of intracellular air-filled vesicles, resulting in cells with positive buoyancy [18]. These vesicles may be purified from the cyanobacteria and used for labeling other cells as they provide contrast for quantitative phase and ultrasonic imaging and MRI [19,20]. Their density, which is that of air surrounded by a thin protein shell, has an average reported value of 0.119 mg/µL [21].

Minerals, in contrast, almost universally have densities much higher than that of water. Very few have specific gravity of less than 1.6, leaving a gap between the density of microorganisms and minerals. Among simple metal oxides, the least dense are lithium oxide (Li_2_O) and silica (SiO_2_), with specific gravities of 2 and 2.08, respectively. Some amorphous forms of silica can have effective densities as low as ~1.6 g/cm^3^ as a result of their processing. Some salts have lower densities: sodium borate decahydrate (Na_2_B_4_O_7_·10H_2_O), magnesium carbonate (MgCO_3_), and carnalite (KClMgCl_2_ (H_2_O)_6_) have specific gravities of 1.7, 1.73, and 1.57 respectively.

Table 1 shows densities for a number of materials of interest for planetary life detection missions, including a line for generic “ocean biominerals,” as described in [22]. The gap between the least dense minerals and the densest bacteria suggests that even a coarse (±~10%) ability to measure particle density can provide valuable insight into whether a passively moving microscopic object is more likely to be a cell or cell-like vesicle or an abiotic mineral. Similar considerations have been used for identification of nanopropulsion in particle solutions [23].

We have previously constructed multiple digital holographic microscopes (DHM) designed for observation of bacteria in aqueous environments [24,25,26]. The instruments are designed with a large observed volume so that large numbers of micrometer-scale objects can be observed simultaneously over time, with up to 15 frame-per-second image acquisition. The nature of the DHM allows us to record the 3D positions of all particles in the field of view at once, without active focus, and then reconstruct time series consisting of image stacks at each time point after the fact. The image stacks can be analyzed for particle positions, and those positions tracked over time to obtain particle trajectories.

The utility of DHM for detection of microorganisms (especially in sparse environmental samples) has been demonstrated for numerous applications, such as diagnostics, water quality measurements, and ocean monitoring of microbial populations and behavior [27,28,29,30,31,32,33,34]. The technique has also been used to investigate the basic biophysics of microbial motility [35,36,37,38,39]. Our research has focused on its applications to astrobiology, using bacterial motility, phase contrast, and in some cases morphology, to discriminate between living and non-living particles [10,25,40,41]. However, classification of non-motile particles at the instrument’s resolution limit remains difficult. The goal of the current study is to aid in disambiguation by observing size- and density-related passive motion of micrometer-sized particles. The particular ability of DHM to observe and record the passive motion of small particles in three dimensions allows us to compare the effects of Brownian motion and gravity to classify particles as potentially cells or minerals based on their density. Unlike typical microscope slide preparations, DHM interrogates a large volume relative to bacterial cells.

In a quiescent liquid volume, motion perpendicular to gravity is driven by Brownian motion, and gives information about particle size and surface properties through the diffusivity. Uniform drift of objects can also be subtracted off, leaving just the Brownian motion. Motion parallel to gravity is driven by a combination of gravity and Brownian motion, where the relative influence of each is determined by the size, surface properties, and density of the particle.

Here we develop a model for particle buoyancy based upon Strouhal number, which is the ratio of the time scales involved in Brownian vs. gravitational motion first described in 1878 [42,43]. We then compare the theoretical model with experimental results obtained using four different micrometer-scale test systems of different buoyancy: alumina beads with negative buoyancy, air-filled cyanobacterial protein vesicles with positive buoyancy, styrene beads with neutral buoyancy, and non-motile bacteria with close to neutral buoyancy. We find that tracking of DHM trajectories shows distinct differences among the different types of particle, with excellent agreement between predicted and observed Strouhal numbers.

## 2. Materials and Methods

### 2.1. Holographic Microscopy

Holographic microscopy was performed using a custom off-axis common path instrument as described previously [24]. Briefly, the paired objective lenses had a numerical aperture (NA) of 0.3, yielding an effective magnification of 19.6×. The wavelength used was 405 nm, supplied by a diode laser (Thorlabs S1FC405); at this wavelength, the microscope’s lateral spatial resolution as measured with an air force test target was 0.8 µm. Samples for recording were diluted into a 1.0 mm deep chamber having both a reference channel (filled with medium or H_2_O) and a sample channel. The chamber was bounded by a microscope slide and coverslip.

Data acquisition was performed using a custom open-source software package, DHMx [44]. All recordings were performed at a frame rate of 15 fps for a total of 20–60 s per recording. Reconstruction in amplitude and/or phase was performed using the angular spectrum method [45] using custom Fiji plug-ins as previously described [46]. Phase reconstructions made use of a reference hologram to remove noise [47]; this reference hologram was the median of all holograms in the recording. The lateral field of view was 356 µm × 365 µm for 2048 × 2048 pixels, and the axial z-spacing was chosen to be 1–2 µm based upon the particle size and nominal axial resolution of the system of 2 µm.

### 2.2. Samples

Aluminum oxide beads were obtained from Micro Abrasives Corp (Westfield, MA, USA, product BG1200). The beads were diluted into phosphate-buffered saline (PBS) to yield 50–100 beads per field of view in any one 2048 × 2048 pixel frame.

Blue-dyed polystyrene microspheres of nominal diameter 1.0 µm were purchased from Polysciences Inc. (Warrington, PA, USA) (Catalog Number 15712) and diluted to a concentration of 10^−4^ M in H_2_O.

Protein-coated air-filled vesicles were purified from the buoyant alga *Anabaena flos-aquae* as previously described [19,20]. The 100 nm-scale vesicles were clustered by first biotinylating with 10^5^ molar excess of EZ-Link-Sulfo-NHS-LC-biotin (ThermoFisher Scientific, Waltham, MA, USA) in PBS for 4 h. After dialyzing twice vs. PBS, the biotinylated vesicles were clustered by incubation with streptavidin (Geno Technology, St. Louis, MO, USA) for 30 min at room temperature at a streptavidin-to-vesicle ratio of 100:1. The resulting clusters were diluted into PBS for recording.

Non-motile bacteria used in this study were *Staphylococcus epidermidis* (ATCC 14990) grown overnight in Lysogeny broth (LB, ThermoFisher Scientific, Waltham, MA, USA) at 37 °C in a shaking incubator to mid-log phase. On the day of the experiment, the culture was diluted into minimal medium (10 mM potassium phosphate, 10 mM NaCl, 0.1 mM EDTA, 0.1 mM glucose, pH 7.0) to 10^6^–10^7^ cells/mL for recording.

### 2.3. Data Processing and Analysis

All manipulations were performed in Fiji [48]. Reconstruction in amplitude or phase produced varying degrees of contrast using the different materials, the selection of which to use was made empirically for each sample.

For the aluminum oxide beads and gas vesicles, holograms were reconstructed in phase, and z-localization was performed using a z-derivative approach as described previously [49].

For the bacteria and polystyrene beads, raw holograms were median-subtracted to increase fringe contrast as previously reported [50]. The holograms were then reconstructed in amplitude.

XY tracking and calculation of the diffusion coefficients were performed using maximum intensity protections of the z-stack at each time point, to create an XYT stack. The plug-in NanoTrackJ [51] was used to determine diffusivity. XZ tracking was performed by reslicing the XYZT hyperstack to create an XZYT hyperstack, then maximum projecting in Y to create an XZT hyperstack. Z velocities were obtained using NanoTrackJ (for bacteria and polystyrene) or TrackMate (for alumina and gas vesicles).

### 2.4. Particle Sizing

The hydrodynamic diameter of the polystyrene microspheres was measured using a DynaPro Plate Reader III (Wyatt Technology, Santa Barbara, CA, USA). Samples were thoroughly mixed prior to analysis. Measurements were made 13 times for the reported hydrodynamic diameter.

The hydrodynamic diameter of the aluminum oxide beads and gas vesicle clusters was measured using a Zeta-PALS (Brookhaven Instruments, Holtsville, NY, USA). Samples were thoroughly mixed prior to analysis. Measurements were made five times for the reported hydrodynamic diameters.

## 3. Results

### 3.1. Model and Predicted Strouhal Numbers

The time scale of Brownian motion is defined by the Einstein–Stokes diffusion coefficient *D*, measured in m^2^/s and given by

(1) $$D=\frac{k_{B}T}{6\pi \eta r},$$

where *k_B_* is Boltzmann’s constant, *T* is the absolute temperature, η is the dynamic viscosity of the medium (1.002 mPa∙s at 20 °C for water), and *r* is the radius of the particle. At 20 °C, this works out to $D=\frac{0.2146}{r}\frac{µm^{2}}{s}.$ In one dimension, a particle will cover an RMS distance *<x^2^>_RMS_* on a time scale

(2) $$\tau_{B}=\frac{{\langle x^{2}\rangle }_{RMS}}{2D},$$

or in two dimensions, a distance *<x^2^+ y^2^>_RMS_*

(3) $$\tau_{B}=\frac{{\langle x^{2}+y^{2}\rangle }_{RMS}}{4D}$$

For gravity, settling or rising is described by Stokes’ Law, which gives a settling velocity *v* that depends upon the difference between the particle density $\rho_{p}$ and fluid density $\rho_{f}$:

(4) $$v=\frac{2\left(\rho_{p}-\rho_{f}\right)gr^{2}}{9\eta }.$$

The timescale needed to traverse a distance *d* is

(5) $$\tau_{g}=\frac{9\eta d}{2\left(\rho_{p}-\rho_{f}\right)gr^{2}}.$$

If the RMS distance and z-distance are both set equal to the particle radius *r*, this gives the Strouhal number *Sr_p_* as

(6) $$Sr_{p}=\frac{\tau_{g}}{\tau_{B}}=\frac{3k_{B}T}{4\pi \left(\rho_{p}-\rho_{f}\right)gr^{4}}.$$

When experimentally determining *Sr_p_*, the diffusivity in *x* and/or *y* may be used to estimate the radius of each particle. Sinking or floating rates in z independent of diffusivity may be used with Equation (4) to get $\rho_{p}-\rho_{f}.$ Because of the high sensitivity of *Sr_p_* to radius, it is important that the measurements be made on each particle independently, since a spread of particle sizes will lead to a significant variation in *Sr_p_* values even within a fairly homogeneous distribution. Gravity and Brownian motion are of equal importance when *Sr_p_*~1. Brownian motion can be ignored for *Sr_p_* << 1; such objects will sink or float rapidly. Note that knowledge of the fluid viscosity is required. For laboratory experiments this can be easily measured or found in tables for known fluids, but for planetary missions a calibration sample with beads of known size and density can be added to the native fluid to obtain viscosity measurements.

**Figure 1:**
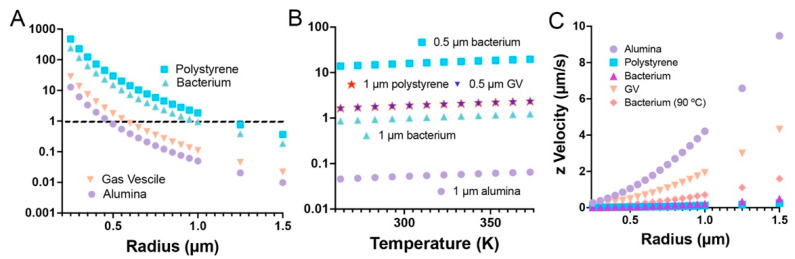
Behavior of typical particles. (**A**) Strouhal number as a function of radius at 20 °C. (**B**) Strouhal number as a function of temperature for selected radii. (**C**) Settling velocity of particles at 20 °C; bacteria at 90 °C added for comparison.

The particles used in this study were found by dynamic light scattering (DLS) to have mean diameters ± 95% C.I. of: polystyrene microspheres, 1944 ± 762 nm; gas vesicle clusters, 690 ± 56 nm; aluminum oxide beads, 1524 ± 470 nm. *S. epidermidis* is spherical and 0.5–1.5 µm in diameter. Assuming density values as in Table 1 gives the predicted *Sr_p_* for the four types of particle across a range of radii as shown in Figure 1A. It can be appreciated from the graph that bacteria and polystyrene beads in the range of our samples will have Strouhal numbers close to 1, indicating that they should be dominated by Brownian motion. Gas vesicle clusters and alumina beads, on the other hand, should show significant sinking (alumina) or floating (vesicles), with the largest particles showing the greatest effect.

Because the Strouhal number and temperature are linearly proportional, the relative differences between the values hold true across all temperatures. This is illustrated in Figure 1B where Strouhal numbers are plotted as a function of temperature for our particles between −10 and 100 °C.

It is important to note that axial resolution is the limiting factor in most volumetric microscopy techniques, so particles must rise or sink enough for the motion to be resolved during the time course of the recording in order to obtain a good value of the Strouhal number. For particles with densities much different from that of water, this does not pose a problem, as expected settling velocities are several micrometers per second in Earth’s gravity. However, for cells and polystyrene, resolving z motion may be difficult. Increasing the temperature to reduce the viscosity of water will increase setting velocities appreciably, as the dynamic viscosity of water is reduced more than 3-fold between 20 °C and 90 °C (from 1.0016 to 0.31417 mPa∙s) (Figure 1C). Conversely, when particles move rapidly, it is important to begin recording immediately after placing the particles into the sample chamber, or they will float or sink to the boundaries of the chamber within seconds.

### 3.2. Experimental Determination of Size and Diffusivity

**Figure 2:**
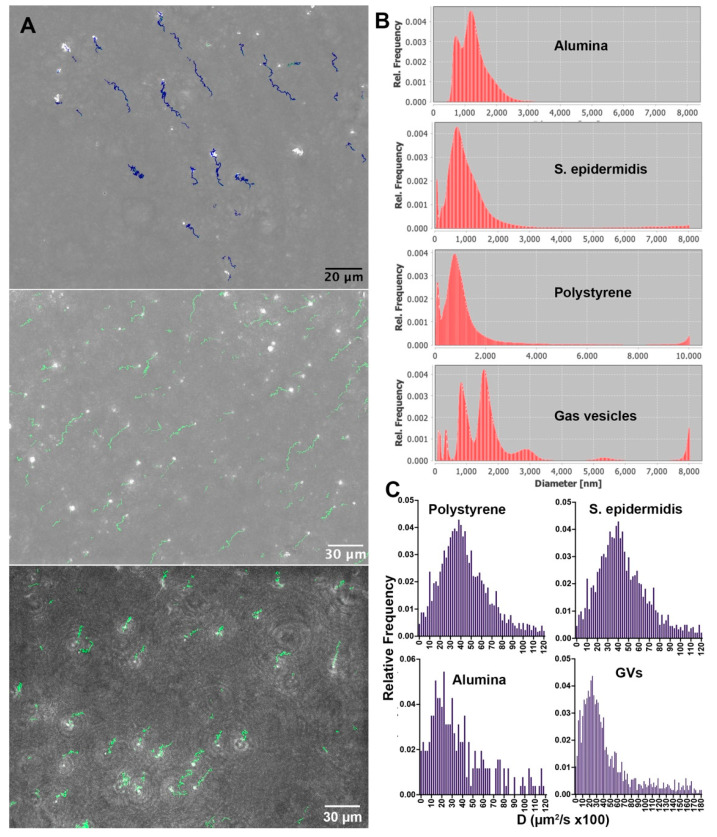
Results from XY tracking. (**A**) Holographic reconstructions: top, maximum intensity phase projection of alumina beads through z, with corresponding tracks over 77 s; middle, maximum intensity phase project of gas vesicles with tracks over 54 s (note the size heterogeneity); bottom; maximum intensity amplitude projection of *S. epidermidis* with tracks over 30 s. There is some degree of XY drift in all images, which is accounted for. (**B**) Size histograms as estimated by the Walker method. (**C**) Diffusion coefficients.

The timescales of Brownian motion may be determined by 2D tracking of XY motion. Figure 2A shows a frame from a tracked dataset of alumina beads, gas vesicles, and *S. epidermidis* with an overlay of identified tracks, with resulting diffusivity and diameter measurements. Videos of particle motion are given in Supplementary Video S1 (alumina), S2 (*S. epidermidis*), and S3 (gas vesicles). Data are shown in Figure 2B,C. Because the distributions of sizes and diffusivities were not Gaussian, histograms are given rather than means, with the median value more representative of the population than the mean. The results agree very well with the predicted Einstein values. Figure 2B shows estimated diameters using the Walker method [52]. The estimated diameter of the alumina beads had two prominent peaks between 1 and 2 µm, consistent with the DLS result. The measured diameters of *S. epidermidis* and the polystyrene beads were unimodal and strongly peaked at 1.0 µm, indicating that these particles did not exhibit significant clustering. The estimated size of the polystyrene beads matched the manufacturer’s specification sheet but was smaller than what we observed using DLS. The gas vesicles showed multiple peaks at increments of 0.8 µm, indicating that significant clusters formed in this sample. The diameter as measured by diffusivity was in excellent agreement with the results found by DLS. Figure 2C shows histograms of diffusion coefficients for the different samples. Statistics of the distributions (N > 100 for all measurements) with outliers > 1.20 µm^2^/s excluded were: *S. epidermidis*: mean ± 95% confidence interval, 0.45 ± 0.02 µm^2^/s; median, 0.41; expected Einstein value, 0.43 based upon r = 0.5 µm; polystyrene, mean ± 95% confidence interval, 0.44 ± 0.02 µm^2^/s; median, 0.40; expected Einstein value, 0.43 based upon r = 0.5 µm; alumina, mean ± 95% confidence interval, 0.37 µm^2^/s; median, 29; expected Einstein value, 28 based upon r = 0.75 µm. For the gas vesicles, only values above 180 × 10^−5^ cm^2^/s were excluded, giving a mean ±95% confidence interval, 44 ± 2 × 10^−5^ cm^2^/s; median, 31. This reflects the clustering of the vesicles that gives the dominant size as >1 µm in diameter.

### 3.3. Particle Axial Motion

**Figure 3:**
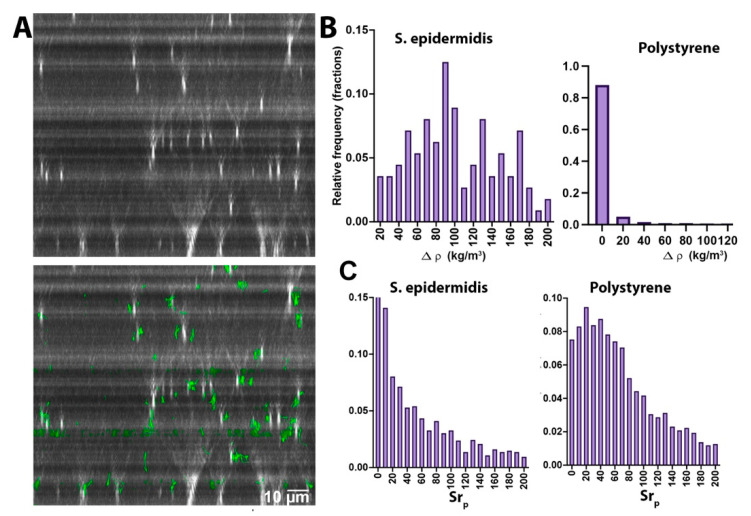
Neutrally buoyant particles. (**A**) *S. epidermidis* visualized in the XZ plane (top) and with tracks overlaid (bottom). (**B**) Calculated density differences vs. water based upon z velocities calculated for *S. epidermidis* and polystyrene, plotted with the same bin widths for easy comparison. (**C**) Strouhal number distributions for *S. epidermidis* and polystyrene.

Tracks without significant z motion indicate that Brownian motion dominates, as in the case of *S. epidermidis* (Figure 3A) or the polystyrene beads (not shown) (see Supplementary Video S4 for a video of *S. epidermidis* XZ motion, and Video S5 for polystyrene). In these cases, NanoTrackJ was used to estimate diffusivity. Any *z* motion would be apparent as drift averaged over all of the particles, rather than on a particle-by-particle basis. The result of this tracking was an overall *z* motion of *S. epidermidis* of 0.2 µm/s. The same procedure was performed for the polystyrene beads, and the measured value over the same time frame was 0.03 µm/s (also see Supplementary Video S5). Figure 3B shows densities $\rho_{p}-\rho_{f}$ calculated from *z* velocities according to Equation (3) for these two types of particles. The mean ± 95% CI value for *S. epidermidis* was 100 ± 8 kg/m^3^, median 93, in excellent agreement with the estimates in Table 1. For polystyrene, most of the measured densities differed from water by less than 20 kg/m^3^, making these beads distinctly different from the bacterial cells. Figure 3C shows Strouhal number distributions for *S. epidermidis* and polystyrene, again illustrating the clear differences.

**Figure 4:**
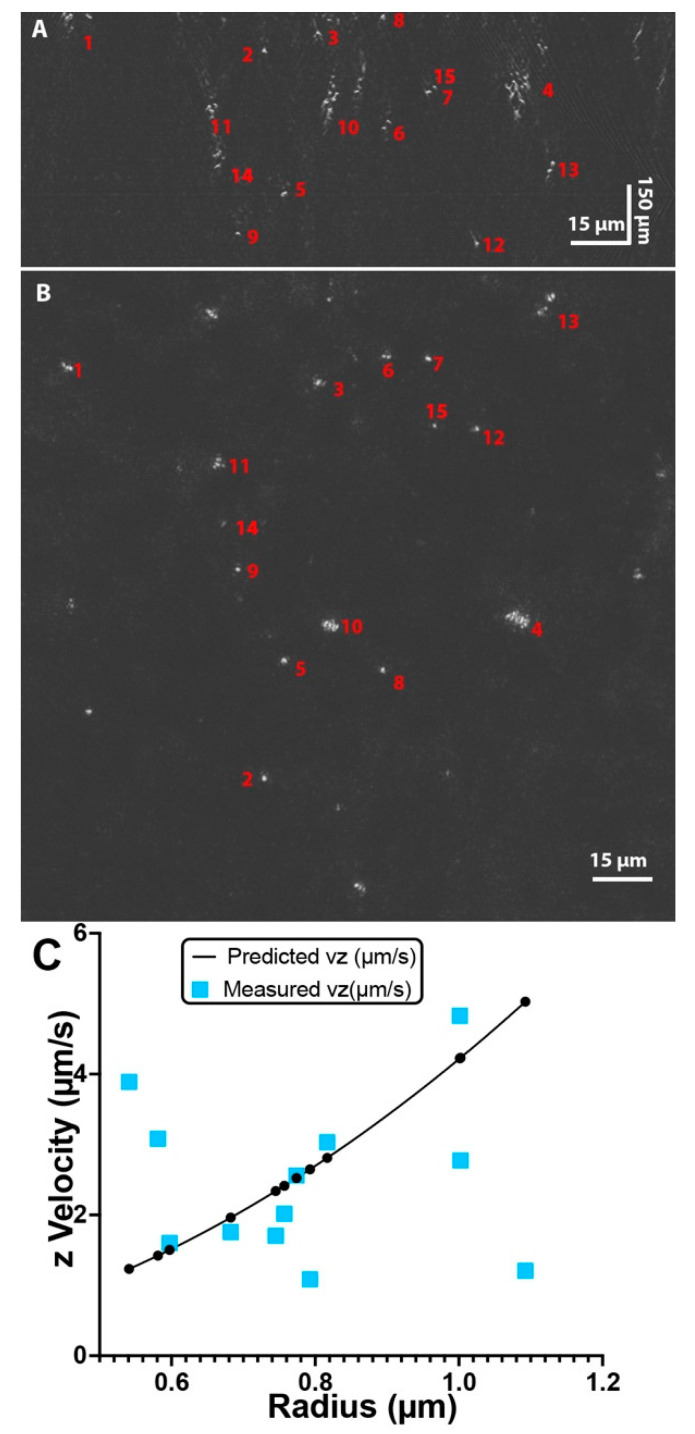
Matching particles in XY and XZ. (**A**) A single time frame of alumina particles in XZ. (**B**) The same time point in XY, showing matched particles according to the X coordinate. (**C**) Relationship of measured z velocities vs. radius (squares) to predicted z velocities (circles).

For alumina, the particles sank or rose in a heterogeneous fashion, so x vs. z motion of individual particles was examined at first rather than taking a mean value. Tracks could be matched in the XY and XZ planes in order to match radii calculated from XY diffusivities with z velocities calculated in XZ (Figure 4A,B). Matching the radii and speeds gave a reasonable relationship of z velocity to radius relative to what was predicted from Equation 4 (Figure 4C). However, the relationship was fairly poor, indicating the stochastic nature of the processes being studied. In order to obtain a better relationship, longer trajectories of single particles would need to be obtained. We thus abandoned this approach and looked at mean values for all of the particles, where averaging over many trajectories substitutes for a single trajectory taken over a long time [53].

**Figure 5:**
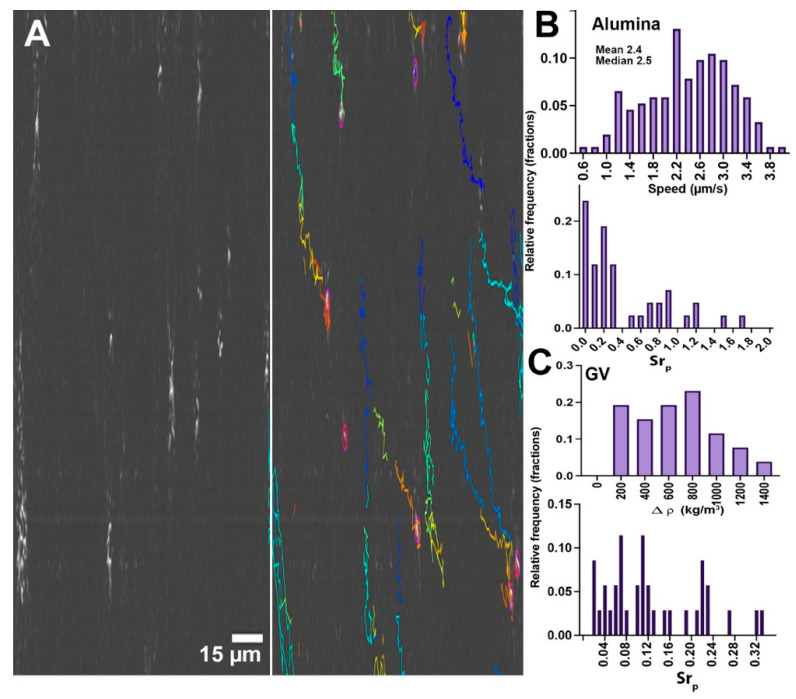
Particles with positive and negative buoyancy. (**A**) Alumina, showing the particles’ appearance in the XZ plane and the tracks over 78 s of time. (**B**) Speed distribution of alumina particles and calculated Strouhal numbers for particles matched in XY and YZ. (**C**) Calculated density difference vs. water for the gas vesicles that showed the highest buoyancy (single peak in the z velocity distribution), and the Strouhal numbers for this subset of particles.

For alumina, the vertical XZT tracks shown in Figure 5A (also see Supplementary Video S6) indicate that sinking dominated in this sample. Particles could be tracked by traditional velocity-based tracking algorithms such as TrackMate. Alumina sank at a rate of 2.4 ± 0.7 µm/s (N = 15), in excellent agreement with the predicted rate (2.36 µm/s for a radius of 0.75 µm, Figure 1). Velocity distributions and Strouhal numbers are given in Figure 5B.

The gas vesicles also showed significant z motion, but floating rather than sinking (not shown) (see Supplementary Video S7). As expected, the velocities of the gas vesicles were multimodal. The most rapid subset had a mean rising velocity of 1.9 µm/s, with a mean radius as measured by diffusivity of 1.0 ± 0.2 µm (median 0.9 µm), in good agreement with the value predicted by the Stokes equation of 1.9 µm/s for r = 1.0 µm. The mean ± 95% CI estimated density difference was 670 ± 130 kg/m^3^. Density values and the calculated Strouhal numbers are given in Figure 5C.

## 4. Discussion

These results illustrate how the instantaneous and volumetric imaging capabilities of DHM allow it to discriminate between non-living and living particles of comparable sizes even in the absence of motility. By analyzing the three-dimensional passive dynamics of beads and non-motile bacteria, the radius and density of the materials may be calculated. Our diffusivities found for *S. epidermidis* are consistent with the Einstein model as well as reported values for other bacteria; for example, non-motile, nonflagellated *E. coli* at room temperature has been reported to have D_X_ = 0.188 μm^2^/s, D_Y_ = 0.154 μm^2^/s [54]. Most bacteria do have some degree of negative buoyancy as well, and we found that 30 s of recording was sufficient to distinguish *S. epidermidis* from polystyrene, which is almost perfectly neutrally buoyant.

**Figure 6:**
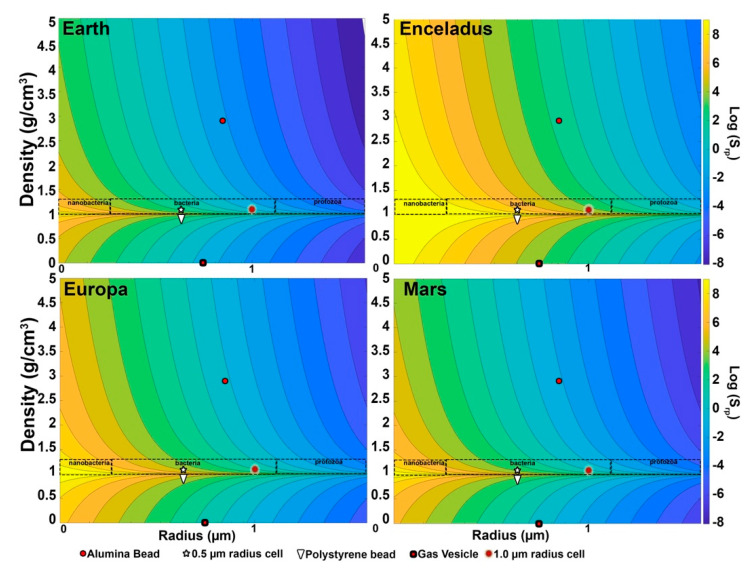
Strouhal numbers of our test samples under the different gravitational environments of selected planetary bodies.

Considering different gravities is important when thinking about space missions. Figure 6 shows log(*Sr_p_*) as a function of particle size and density for four different gravity environments of interest to the search for extant life: Earth, Europa, Enceladus, and Mars. These span roughly two orders of magnitude of gravity, but careful examination shows common features that can be used to aid in classifying particles. For terrestrial species and minerals, there is a gap between ~1.3 and 1.5 g/cm^3^ density that helps distinguish cells or vesicles from minerals, so that even a ~10% uncertainty in density will leave a clear separation. Terrestrial cells in aquatic environments have densities consistently less than 1.3 g/cm^3^ (with most less than 1.2 g/cm^3^) and very few minerals have densities as low as 1.5 g/cm^3^. This is particularly useful if the size of the object can also be determined as above, as *Sr_p_* for cell-like objects is 3× to 5× *Sr_p_* for the lightest minerals.

On each body, the motion of particles with a radius larger than ~1 µm is gravity dominated and their density can be estimated from Stokes’ Law (Equation (3)). Though Stokes’ Law results are affected by geometry and surface properties of particles, objects in this size range are also resolvable by the microscopes described earlier and their size and shape can be directly measured and included in the classification process. At lower gravities and smaller sizes in this range, particles with densities in the cellular range are governed by both Brownian motion and gravity, and this type of mixed behavior is an indication for large particles that they are likely to be cell-like, as described below.

Below ~1 µm and down to 0.4 to 0.7 µm, depending on the gravity environment, particles generally fall into the Brownian-dominated or the mixed region. Within the mixed region, measurement of the diffusion constant can be used to determine particle size accurately, even below the optical resolution of the microscope. Sufficiently long observations can be used to measure the velocity well enough to obtain the difference in density from the surrounding fluid. On Earth, only 30 s of observation were required to distinguish bacteria from styrene beads by their buoyancy, a density difference of ~0.05 g/cm^3^. If we only require resolution of ~0.1 g/cm^3^, the time necessary is about 15 s. Stokes’ Law is linear in *g*, so on Europa or Mars the observation time necessary to distinguish minerals from cell-like objects would only be a few minutes. On Enceladus, with only ~1% of Earth’s gravity, this would require on the order of 1500 s, which might also impose requirements on the stability of the lander system during that time. Additional analyses, including index of refraction measurements from the DHM and fluorescence imaging, could be used to classify these objects.

## 5. Conclusions

We have shown that the relative effects of Brownian motion and gravity can be measured with existing instruments to discriminate densities of objects of sizes and in gravitational environments that are of interest for life detection. While this approach does not directly determine if something is alive or not, it can provide strong support for whether an object is cell-like or mineral-like based on its density, with sensitivity to distinguish bacteria from neutrally buoyant polymers. When combined with other data that can be obtained simultaneously in a microscope (size, structure, refractive index, autofluorescence, stained fluorescence) it can strengthen the classification of whether a particular object is likely to be a live cell or inorganic mineral grain. This analysis adds to the toolbox for DHM analyses of samples containing bacteria and microminerals. Previous studies have indicated that DHM is an excellent technique for detection and classification of motile organisms, but that when microorganisms are non-motile and near the resolution limit, there is insufficient phase contrast or morphological information to confidently distinguish prokaryotes from microminerals. These new techniques may help to classify objects at the resolution limit as potentially biological or abiological. Future work will apply these methods to field samples containing unfiltered water samples and to other strains and life-cycle stages of bacteria, including those under heat or cold stress and toxin exposure.

## Figures and Tables

**Table 1:** Density of various materials is the difference between the material and water at 20 °C.

Material	Density (kg/m^3^)	(kg/m^3^)
Water	998 at 20 °C	0
Bacterial Cell	1100	102
Diatom Cell	1100–1200	102–202
Carnalite	1570	572
Suspended Ocean Minerals	1600	602
Borax (Decahydrate)	1700	702
Silica	2080	1082
Calcium Carbonate	2710	1712
Alumina	2930	1932
Polystyrene	1050	52
*Anabaena* Gas Vesicles	119	−879

## Data Availability

The raw holograms for the datasets used here are deposited in a public depository at Data Dryad, https://doi.org/10.5061/dryad.n02v6wwxj (accessed on 5 August 2021). Other data are available from the authors upon request.

## Supplementary Materials

The following are available online at https://www.mdpi.com/article/10.3390/life11080793/s1, Video S1: alumina bead phase reconstruction in XY over 60 s; Video S2: *S. epidermidis* amplitude reconstruction in XY over 30 s; Video S3: gas vesicle phase reconstruction in XY over 60 s; Video S4: *S. epidermidis* XZ plane over 30 s with identified tracks; Video S5: Polystyrene XZ plane over 30 s; Video S6: alumina beads over 77 s in XZ; the Z scale is 10 times the X scale; Video S7: gas vesicles in in XZ over 60 s.
